# Familial hypercholesterolemia in children and the importance of early treatment

**DOI:** 10.1097/MOL.0000000000000926

**Published:** 2024-02-14

**Authors:** Sibbeliene E. van den Bosch, Barbara A. Hutten, Willemijn E. Corpeleijn, D. Meeike Kusters

**Affiliations:** a Amsterdam UMC location University of Amsterdam, Department of Pediatrics; b Amsterdam Cardiovascular Sciences, Diabetes and Metabolism; c Amsterdam Gastroenterology Endocrinology Metabolism; d Amsterdam UMC location University of Amsterdam, Department of Epidemiology and Data Science, Meibergdreef 9, Amsterdam, The Netherlands

**Keywords:** atherosclerotic cardiovascular disease, children, familial hypercholesterolemia, screening, treatment

## Abstract

**Purpose of review:**

Familial hypercholesterolemia leads to elevated levels of low-density lipoprotein cholesterol (LDL-C) from birth onwards due to a pathogenetic variation in genes in cholesterol metabolism. Early screening to identify and subsequently treat children with familial hypercholesterolemia is crucial to reduce the risk of premature atherosclerotic cardiovascular disease (ASCVD). This review focuses on recent insights in the field of pediatric familial hypercholesterolemia.

**Recent findings:**

Screening in childhood and early initiation of optimal lipid-lowering therapy (LLT) have shown promising outcomes in the prevention of ASCVD. In addition, cost-effectiveness research has demonstrated highly favorable results. With the availability of novel therapies, familial hypercholesterolemia has become a well treatable disease.

**Summary:**

Children with familial hypercholesterolemia benefit from early detection and optimal treatment of their elevated LDL-C levels.

## INTRODUCTION

Familial hypercholesterolemia is the most common metabolic disease caused by a genetic variation in one of the genes involved in cholesterol metabolism [1]. Clinically, patients with familial hypercholesterolemia are characterized by elevated LDL-cholesterol (LDL-C) levels from birth onwards, which results in an increased risk of premature atherosclerotic cardiovascular disease (ASCVD) [2]. Without adequate lipid-lowering therapy (LLT), patients with the heterozygous form of familial hypercholesterolemia (HeFH, one allele affected, prevalence 1 : 311 individuals) with an LDL-C level more than 5.5  mmol/l have a 20-fold higher risk to develop ASCVD compared with unaffected adults with an LDL-C level less than 3.5 mmol/l [2–4]. Regarding the more severe homozygous form of familial hypercholesterolemia (HoFH, two alleles affected, prevalence 1 : 400 000 individuals), in some cases, children develop cardiovascular disease by the age of 10 without lipid-lowering therapy [5]. This review focuses on recent insights in the field of early screening and treatment of children with familial hypercholesterolemia. 

**Box 1:**
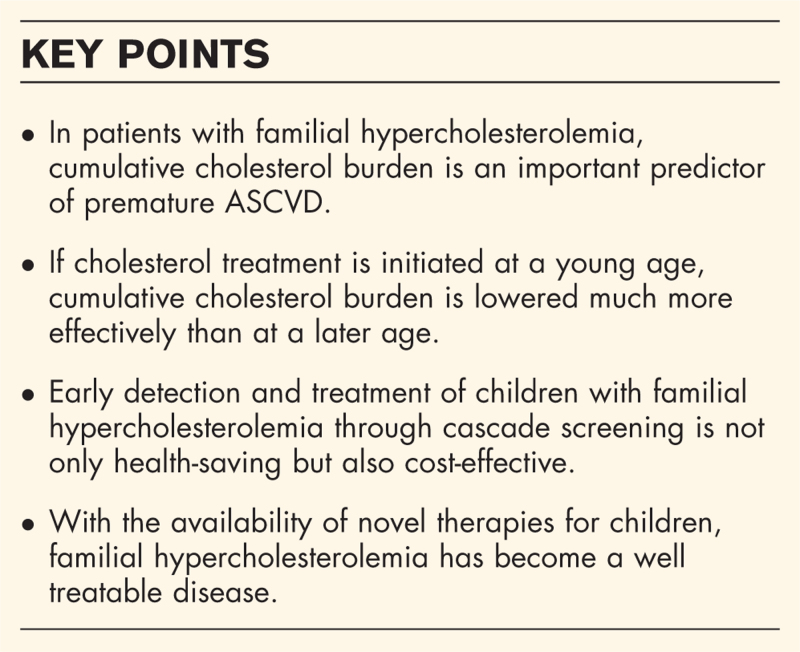
no caption available

### Case scenario: father and son

*An 8-year-old boy is referred to a pediatrician for treatment of his hypercholesterolemia (LDL-C 6.1 mmol/l). His father, 47 years old, has xanthelasmata on both eyelids and is known with hypercholesterolemia (LDL-C 8.2 mmol/l) but does not use his lipid-lowering medication. Mother has an untreated LDL-C level of 1.8 mmol/l. As far as known, there have been no cases of ASCVD on either the maternal or paternal side of the family. Genetic testing for familial hypercholesterolemia has not been conducted in the family. For several years, father has been experiencing chest pain, and cardiovascular imaging showed the presence of coronary plaques. How can we ensure that his 8-year-old son will age healthy?*

## CUMMULATIVE CHOLESTEROL BURDEN AND ATHEROSCLEROTIC CARDIOVASCULAR DISEASE

**FIGURE 1:**
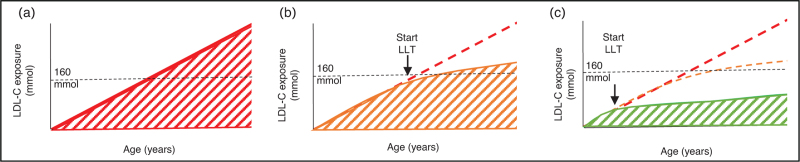
Schematic illustration of cumulative LDL-C exposure in three patients. (a) A patient with FH who is not treated with lipid-lowering therapy. Cumulative LDL-C exposure increases with age and significantly surpasses the 160 mmol threshold for the onset of ASCVD. (b) A patient with FH who has been treated with LLT since adulthood. At the time of therapy initiation, the patient already had a substantial exposure to LDL-C. After initiating LLT the increase in LDL-C exposure is mitigated. (c) A patient with FH who has been optimally treated with LLT since childhood. The cumulative LDL-C exposure is clearly less elevated compared to patients A and B. LDL-C, LDL cholesterol; LLT, lipid-lowering therapy.

A significant determinant in assessing health risks for patients with familial hypercholesterolemia is the concept of ’cumulative cholesterol burden’, which represents the cumulative LDL-C exposure to the arterial wall within a specific time frame. Results of a recent study among 188 adults with familial hypercholesterolemia showed that cumulative cholesterol burden play a more significant predictor role in the development of ASCVD in patients with familial hypercholesterolemia compared to total cholesterol levels or LDL-C levels alone [6^▪▪^]. In another study also, the impact of cumulative cholesterol burden on coronary plaque burden was assessed and the results showed that every 75* *mmol of LDL-C exposure was associated with a doubling in coronary plaque volume [7]. A commonly used threshold of 160* *mmol is considered the point at which ASCVD are likely to manifest [8]. As seen in Fig. 1, cumulative cholesterol burden can be reduced much more effectively if lipid-lowering therapy (LLT) is initiated in childhood instead of in adulthood. Therefore, current guidelines recommend the initiation of pharmacological LLT in children with HeFH at the age of 8 if lifestyle recommendations (exercise, healthy food) do not achieve the target LDL-C levels (≥8 years LDL-C <4.0 mmol/l, ≥10 years LDL-C <3.35 mmol/l). In children with HoFH, treatment is needed as early as possible. To illustrate, if the LDL-C levels of a child with HoFH is 20 mmol/l, without treatment, this child would reach the 160* *mmol target already at the age of 8 years [9].

## EVIDENCE OF EFFICACY AND SAFETY OF EARLY TREATMENT

**FIGURE 2:**
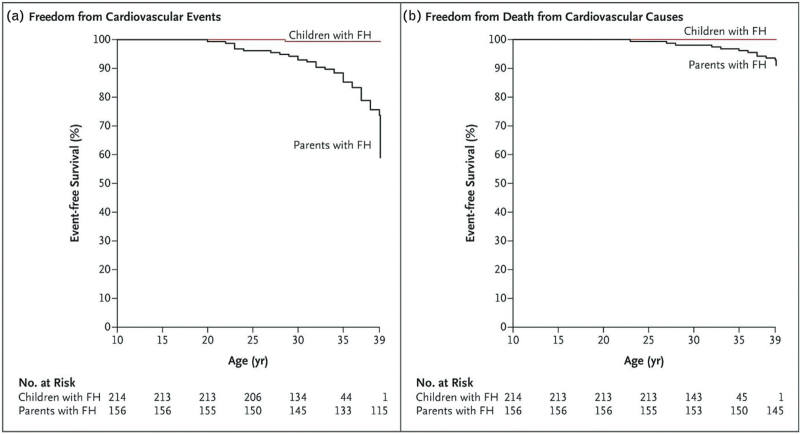
Kaplan-Meier curves for patients with familial hypercholesterolemia who began receiving statin therapy during childhood and their affected parents for whom statins were available much later in life. These results support the aforementioned statements emphasizing the critical importance of early detection and treatment. Adapted from [12]. FH, heterozygous familial hypercholesterolemia.

Studies have been conducted in children with familial hypercholesterolemia to investigate both the short and long-term effectiveness and safety of statins, including during puberty [10,11]. A prospective cohort study from the Netherlands includes 214 children with HeFH and their 95 unaffected siblings. After 20 years of follow-up, it was shown that progression of carotid intima-media thickness (cIMT), a surrogate marker of atherosclerosis, in children with HeFH who were treated with LLT normalized and was similar to that in their unaffected siblings (mean difference adjusted for sex, −0.0001 mm per year; 95% confidence interval, −0.0010 to 0.0008). Additionally, it was observed that the cumulative incidence of cardiovascular events and of death from cardiovascular causes before the age of 40 was lower amongst children with HeFH who were early treated with LLT, than amongst their parents with familial hypercholesterolemia (Fig. 2) [12]. A next follow-up assessment of this cohort will be highly valuable as these children with HeFH will have surpassed the age of 40, which is when ASCVD is frequently observed in patients with familial hypercholesterolemia due to their cumulative cholesterol exposure.

## CONVENTIONAL AND NOVEL LIPID-LOWERING THERAPIES

### Heterozygous familial hypercholesterolemia

**FIGURE 3:**
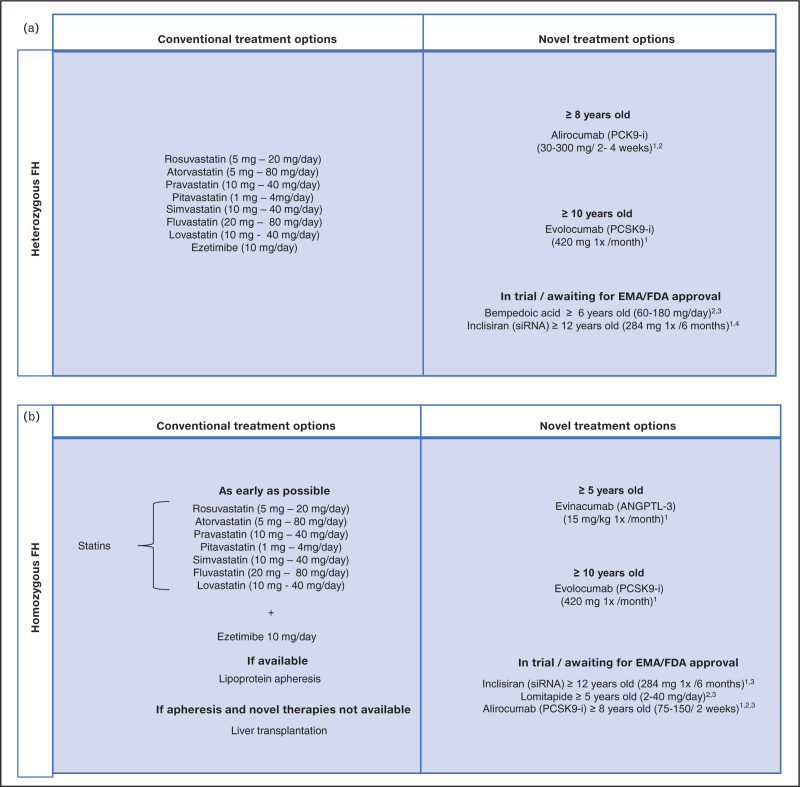
(a) Overview of conventional and novel treatment options for children with heterozygous familial hypercholesterolemia. ^1^Subcutaneous injection. ^2^Based on weight ^3^phase II trial ^4^phase III trial. PSCK9-i, proprotein convertase subtilisin/kexin type 9 inhibitor; siRNA, small interfering ribonucleic acid. (b) Overview of conventional and novel treatment options for children with homozygous FH ANGPTL-3, angiopoietin-like 3; PSCK9-i, proprotein convertase subtilisin/kexin type 9 inhibitor; siRNA, small interfering ribonucleic acid. ^1^Subcutaneous injection. ^2^Based on weight/age. ^3^Phase III trial.

**FIGURE 4:**
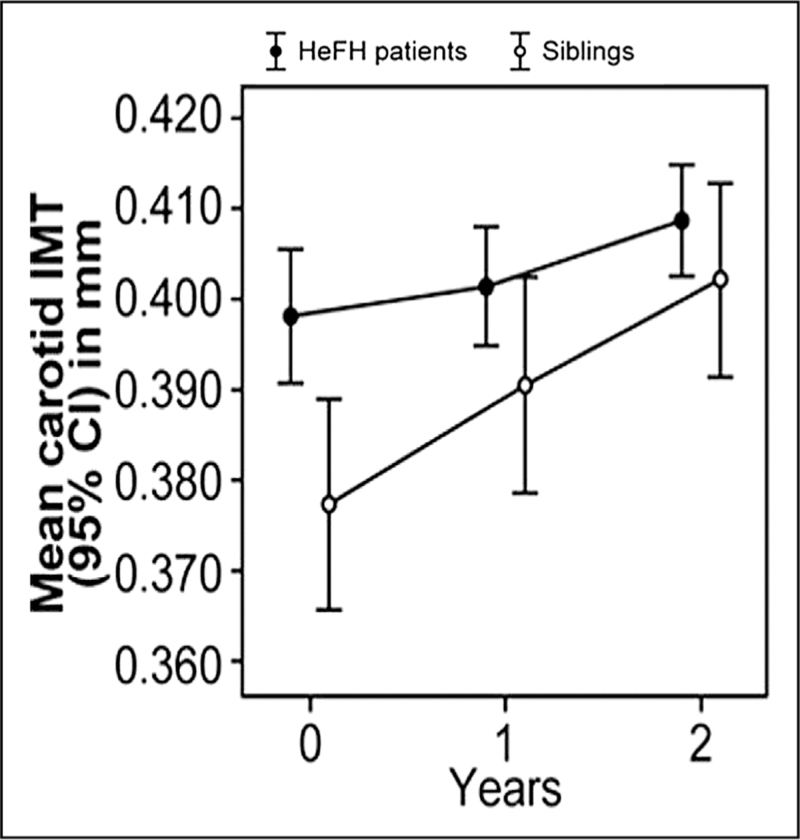
Less progression of carotid intima-media thickness in children with heterozygous familial hypercholesterolemia versus their unaffected siblings after 2 years treatment with rosuvastatin from Braamskamp *et al.*[19].

As a result of worldwide advancements in research in the pediatric field of familial hypercholesterolemia, there are currently more and more LLTs available and in trial to effectively treat both children with HeFH and HoFH [13]. In addition to conventional oral LLT like statins and ezetimibe, novel therapies are available if these are not sufficient to reach treatment targets or are unsuitable due to side effects. Research on statin use in 371 adolescents and children showed that 30% of young familial hypercholesterolemia patients had poor adherence to statins [14]. Although the main reason for nonadherence in this study was lack of motivation in which focus on patient engagement can help, some children were intolerant for statins. Statin intolerance in children has not been extensively studied and the incidence is unknown. From our experience with the before mentioned trial and what we see in the daily practice, statins are generally very well tolerated in youth [12]. However, a well known side effect of statins is myopathy, with or without creatine kinase elevation. One of the causes for statin intolerance might be a genetic determinant, such as single-nucleotide polymorphisms in SLCO1B1, but literature is inconclusive and genetic testing is not recommended for statin intolerance diagnosis [15]. In case of statin intolerance, adjusting statin dosage or switching to another statin is recommended. If that does not solve it and there are still substantial side effects, or if LDL-C targets are not achieved due to high LDL-C levels, novel treatment options can be valuable for these children, including proprotein convertase subtilisin/kexin type 9 (PCSK9-i) or bempedoic acid (currently in trial). An overview of currently available medication for children with HeFH is shown in Fig. 3a. Children with HeFH are not routinely screened for subclinical atherosclerosis as part of clinical follow-up. In research settings, carotid intima-media thickness (cIMT) is determined using carotid ultrasound. However, the clinical significance of cIMT is not proven at the individual level, but is highly informative in a research context. Several studies have shown that children with HeFH have a higher mean cIMT, measured by ultrasound, compared to their unaffected siblings [16,17]. In addition, a recent meta-analysis (2022) showed, based on 6000 individuals, treated HeFH patients have a smaller mean cIMT (95% CI) difference with controls (measuring 0.05 (0.03–0.08) mm (*P* < 0.001)) versus untreated HeFH patients and controls [0.12 (0.03–0.21) mm (*P* = 0.009)] [18]. In a study focusing on cIMT, it was found that after 2 years treatment with rosuvastatin (daily 5–10 mg), there was significantly less progression of the mean carotid IMT and a decrease of the maximum cIMT of the increased cIMT in children with familial hypercholesterolemia compared to their untreated unaffected siblings (Fig. 4) [19]. These results align with a prior trial in children with HeFH who were treated for 2 years with pravastatin (20–40 mg/day) and showed a cIMT regression (mean [SD], −0.010 [0.048] mm; *P* = .049), familial hypercholesterolemia children with HeFH who received a placebo (mean [SD], +0.005 [0.044] mm; *P* = 0.28) showed a cIMT progression [20]. These findings provide support for the hypothesis that early treatment is beneficial [18]. A subgroup within the children with HeFH, the severe HeFH children (LDL >90th percentile for age and sex among familial hypercholesterolemia patients) have a cumulative cholesterol exposure that is comparable to that of HoFH children [21]. However, treatment guidelines and recommendations for assessment of subclinical atherosclerosis are based on (genetic) diagnosis, HeFH or HoFH, and not on cumulative cholesterol exposure. This raises the question if the subgroup of children with severe HeFH should be treated differently than children with ‘mild’ familial hypercholesterolemia. Therefore, we performed CTCA in children with severe HeFH. In this small study involving four children, no atherosclerotic abnormalities were found despite the high cumulative cholesterol exposure. However, this was a very limited study group, and further research is needed to determine whether additional imaging is indeed unnecessary for these severe HeFH children [22]. Until more focused guidelines are available for these children, shared decision-making between physician, parents and child is needed. Based on factors such as potential cardiac symptoms, age of diagnosis, severely increased LDL-c levels, coincident high lipoprotein(a) levels or severe pathogenic genetic familial hypercholesterolemia variant, it can be individually argued if screening for subclinical atherosclerosis is necessary.

### Homozygous familial hypercholesterolemia

Developments in LLT are especially crucial for children with HoFH as they, without early and optimal treatment, develop cardiovascular disease in childhood [5]. As conventional LLT (statins, ezetimibe) are mostly insufficient for these children, they are dependent on lipoprotein apheresis, a technique of recurrent extracorporeal removal of lipoproteins, if locally available. PCSK-9 inhibitors are also effective for children with HoFH. However, due to their mechanism of action, which relies on residual LDL-R activity, these medications are not effective in children with a null-null pathogenic genetic variant. Evinacumab, a novel treatment option for children with HoFH functions independently of residual LDL-R activity. This intravenous human mAb inhibits angiopoietin-like 3 (ANGPTL3), another key protein involved in lipid metabolism showed very promising results: After 6 months of treatment with evinacumab in two children (aged 12 and 16 years) not only LDL-C levels were diminished, also a regression of atherosclerotic plaque was seen [23]. This suggests that atherosclerosis may be reversible in people with HoFH as long as the plaque is not calcified yet. An overview of currently available medication for HoFH is shown in Fig. 3b. In children with HoFH, screening for subclinical atherosclerosis is of great importance, because findings can lead to intensification of adjustment of therapy. At present, the recommendation is to perform echocardiography every year and coronary computed tomography angiography (CTCA) from the age of 3 and follow up if clinically indicated [24]. In a cross-sectional study of children with HoFH who underwent apheresis treatment from the age of 10, signs of atherosclerosis in the form of calcifications were observed in all of them on CTCA [25]. Next to CTCA, the use of artificial intelligence may also play a valuable role in assessment of atherosclerosis in the near future [26].

## DIAGNOSIS AND SCREENING FOR FAMILIAL HYPERCHOLESTEROLEMIA IN CHILDREN

Diagnosing familial hypercholesterolemia already in childhood is of paramount importance as this provides the only possibility to start LLT early enough to really mitigate AVSCD risk to levels comparable with the general population. Unfortunately, patients with familial hypercholesterolemia are generally diagnosed in adulthood [median age (IQR) 44.4 years (32.5–56.5)] and are often under-treated [27]. Screening children for familial hypercholesterolemia can be done in various proven effective ways. Firstly, testing for familial hypercholesterolemia in children can be conducted in children who, based on their family history or high lipid levels, are suspected for familial hypercholesterolemia. A child is suspected of having HeFH when LDL-C levels at least 5.0 mmol/l on two successive occasions or an LDL-C at least 4.0 mmol/l with a positive family history of ASCVD or an LDL-C at least 3.5 mmol/l with an parent with HeFH [9]. In all cases, it is important to rule out other reasons for hypercholesterolemia such as hypothyroidism, nephrotic syndrome and obesity [9]. Current guidelines recommend to test children who are suspected of HeFH at the age of 5, and children suspected for HoFH need to be tested as early as possible to start treatment immediately [9]. In the current guidelines, the untreated LDL-C levels of children suspected for HoFH have recently been revised from 13.0 to 10.0 mmol/l [24]. In addition, children can be clinically diagnosed with HoFH if they have a treated LDL-C level more than 8.0 mmol/l and presence of xanthoma before the age of 10 or an increased LDL-C and presence of HeFH in both parents [9].

Secondly, testing in children can be conducted through cascade screening via parents as children of patients with familial hypercholesterolemia have 50% chance of having FH. In the Netherlands, over 28 000 patients with familial hypercholesterolemia were identified due to a successful national cascade screening program (active during the period 1994–2014) [28].

A third successful option for detecting children with familial hypercholesterolemia is universal screening. In Slovenia, since 2011, all children have undergone screening for familial hypercholesterolemia at the age of 5 years using total-cholesterol testing. If there is a suspicion of familial hypercholesterolemia based on this screening, these children undergo genetic testing [29].

The diagnosis familial hypercholesterolemia can be made based on clinical criteria or by genetic testing to provide a definite molecular diagnosis. If locally available, molecular genetic testing is recommended using Sanger sequencing or next-generation sequencing- based custom packages [13]. Genetic analysis can give additional insight in the severity of the specific genetic variation causing familial hypercholesterolemia and in the case of HoFH, give information on whether or not LDL receptor based therapies are an option for the individual patient [13]. A recent (2023) retrospective study from the Netherlands based on 2714 genetic test results from children with HeFH demonstrated that children having one LDL-R null genetic variant, wherein LDL-receptors exhibit no activity, have significantly higher LDL-C levels when compared to those with an LDL-R defective allele with rest activity (6.0 versus 4.9 mmol/l, *P* < 0.001). A similar line was observed in the children with HoFH (*n* = 41), wherein a greater number of null alleles corresponded to higher LDL-C levels. In the implementation of all forms of screening, the cost-effectiveness is a crucial aspect. Several studies have been conducted to investigate the cost-effectiveness of screening for familial hypercholesterolemia in childhood. A recent cost-effectiveness study on the identification of familial hypercholesterolemia in children and the start of start treatment with statins (mean age 10 years) showed that detection and treatment for children were both health and cost saving compared to no cascade screening [30^▪▪^]. This aligns with the results of a previous Australian study in which 148 children from 126 parents with familial hypercholesterolemia were genetically tested for familial hypercholesterolemia concluding that that cascade testing is effective and can contribute to early initiation of statin therapy, leading to significant reductions in LDL-C levels [31]. It is of great importance that medical doctors are keen to test and treat children with or suspected for familial hypercholesterolemia. Pediatricians play a crucial role in screening and treatment of children with familial hypercholesterolemia. Recently we conducted a survey amongst pediatricians in the Netherlands to explore potential gaps in knowledge on familial hypercholesterolemia care for children (S.E. van den Bosch, W.E. Corpeleijn, S. Ibrahim, *et al*., in preparation). We found alarming gaps in awareness and knowledge on familial hypercholesterolemia care in children and these gaps may lead to suboptimal detection and treatment of children with familial hypercholesterolemia. Therefore, actions are required to close these gaps in order to prevent ASCVD at a later age in patients with familial hypercholesterolemia.

### Back to the case scenario: father and son

*Genetic testing revealed that both father and the son have one pathogenetic variant of the LDL receptor, confirming the genetic diagnosis of heterozygous familial hypercholesterolemia. In the 8-year-old boy, we initiated 5 mg of rosuvastatin daily, resulting in a decrease in LDL-C from 6.1 to 4.1 mmol/l in 6 weeks. Subsequently, the rosuvastatin dose was increased to 10 mg per day, leading to a further reduction in LDL-C to 3.1 mmol/l, which is below the target value of 4.0 mmol/l for his age (<10 years). No side effects occurred. We have scheduled a follow-up appointment for him in one year at our pediatric lipid clinic. Father was motivated to take his lipid-lowering medication, and an appointment with his lipidologist was made.*

## CONCLUSION

Early screening and subsequently optimal treatment of children with familial hypercholesterolemia has been proven to be well tolerated, effective in reducing cardiovascular risk and cost-effective. With the availability of novel therapies, familial hypercholesterolemia has become a well treatable disease. Further long-term follow-up studies are eagerly wanted to determine if early screened children familial hypercholesterolemia with adequate LLT, including children with the severe homozygous form of familial hypercholesterolemia (HoFH), can all grow old free from ASCVD.

## Acknowledgements

*None.*

### Financial support and sponsorship

*B.A.H. received a research grant from Silence Therapeutics. All other authors declare no competing interests.*

### Conflicts of interest

*There are no conflicts of interest.*

## REFERENCES AND RECOMMENDED READING

Papers of particular interest, published within the annual period of review, have been highlighted as:

▪ of special interest
▪▪ of outstanding interest
